# Phylogeography of an endangered disjunct herb: long-distance dispersal, refugia and colonization routes

**DOI:** 10.1093/aobpla/ply047

**Published:** 2018-08-21

**Authors:** Javier Bobo-Pinilla, Julio Peñas de Giles, Noemí López-González, Sonia Mediavilla, M Montserrat Martínez-Ortega

**Affiliations:** 1 Departamento de Botánica, Universidad de Salamanca, Salamanca, Spain; 2 Banco de ADN vegetal, Banco Nacional de ADN, Salamanca, Spain; 3 Unidad de Conservación Vegetal, Departamento de Botánica, Universidad de Granada, Granada, Spain; 4 Departamento de Biología Animal, Parasitología, Ecología, Edafología y Química Agrícola, Universidad de Salamanca, Salamanca, Spain

**Keywords:** AFLP, *Astragalus edulis*, LDD, palaeogeographical models, phylogeography, plastid DNA, western Mediterranean, Macaronesian area

## Abstract

Quaternary glacial cycles appear to have had a consistent role in shaping the genetic diversity and structure of plant species. Despite the unusual combination of the characteristics of the western Mediterranean–Macaronesian area, there are no studies that have specifically examined the effects of palaeoclimatic and palaeogeographic factors on the genetic composition and structure of annual herbs. *Astragalus edulis* is a disjunct endemic found in the easternmost Canary Islands and the semi-arid areas of north-eastern Africa and south-eastern Iberian Peninsula. This endangered species shows no evident adaptations to long-distance dispersal. Amplified fragment length polymorphism (AFLP) data and plastid DNA sequences were analysed from a total of 360 individuals distributed throughout the range of this species. The modelled potential distribution of *A. edulis* under current conditions was projected over the climatic conditions of the Last Interglacial (130 ka BP) and Last Glacial Maximum (21 ka BP) to analyse changes in habitat suitability and to look for associations between the modelling and genetic results. Amplified fragment length polymorphism analysis showed clear phylogeographic structure with four distinct genetic clusters. Approximate Bayesian computation (ABC) models based on plastid DNA sequences indicated a Middle Pleistocene long-distance dispersal event as the origin of the populations of the Canary Islands. The models also suggested south-western Morocco as the ancestral area for the species, as well as subsequent colonization of north-eastern Morocco and the Iberian Peninsula. The data compiled indicated the possibility of the presence of refuge areas at favourable locations around the High Atlas and Anti-Atlas mountain ranges. Moreover, palaeodistribution models strongly support the events inferred by ABC modelling and show the potential distribution of the species in the past, suggesting a putative colonization route.

## Teaser

*Astragalus edulis* is a disjunct endemic of the western Mediterranean–Macaronesian area. Amplified fragment length polymorphism analysis showed clear phylogeographic structure. Approximate Bayesian computation models based on plastid DNA sequences indicated a Middle Pleistocene long-distance dispersal event as the origin of the populations of the Canary Islands. The modelled potential distribution of *A. edulis* under current conditions was projected over the climatic conditions of the Last Interglacial (LIG) and Last Glacial Maximum (LGM) to analyse changes in habitat suitability. The models suggested south-western Morocco as the ancestral area and the presence of a putative colonization route along the south of the Atlas Mountains to the Northeast of Morocco during the LGM.

## Introduction

Current diversity patterns are influenced by both historic and recent environmental conditions. Northern Hemisphere phylogeography relies on the idea that Quaternary glacial/interglacial cycles affected the distribution of plant communities and species (Weiss and Ferrand 2007). As a result, the nature of colonization and settlement patterns after the last glacial period is of particular interest to conservation (Soliani *et al.* 2015). Investigating the possible historical dispersal routes of endangered species, with relatively wide and fragmented distribution areas, may provide useful information for the effective implementation of affordable conservation measures.

The Mediterranean basin represents a crossroad for plant migration, being a centre of active speciation and a major Pleistocene refugium (Terrab *et al*. 2008b; Médail and Diadema 2009; and references therein). The western Mediterranean–Macaronesian transition area bears an unusual combination of characteristics, which includes a geographical closeness between continents and between oceanic islands and mainland areas, as well as a broad range of geological ages, palaeoclimatic events and palaeogeographic features. A pre-eminent characteristic of oceanic islands is that they furnish clear-cut spatial and temporal limits and therefore act as living laboratories for studies on the effects of historical colonization, dispersal, geographical isolation and other evolutionary patterns of plants (e.g. Fernández-Mazuecos and Vargas 2011; Lo Presti and Oberprieler 2011; and references therein).

**Figure 1.**
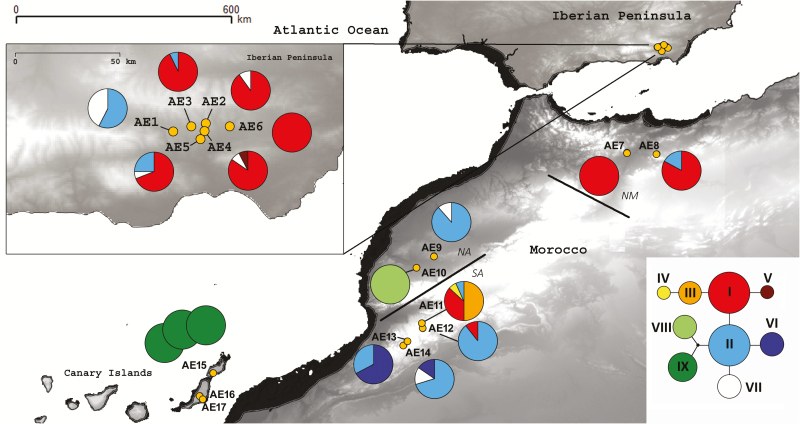
Sampling locations covering the present distribution of *Astragalus edulis*. Coast lines during the LGM (black shadow). Plastid haplotype distribution of the species; plastid haplotype network (circle size is proportional to the number of individuals for each haplotype). Clustering for the DIYABC analysis labelled (Iberian Peninsula; northern Morocco, NM; northern Atlas, NA; southern Atlas, SA; Canary Islands).

Several authors have proposed that the Mediterranean region has been the main floristic source for dispersal and diversification of new evolutionary lineages in Macaronesian islands (Marrero 2004; Vargas 2007). Numerous molecular studies on Canary Island flora suggest that geographic isolation and colonization between islands, with similar ecologic characteristics, have been strong driving forces for the diversity found within the Canary archipelago (Francisco-Ortega *et al.* 1996; Marrero 2004; Fernández-Mazuecos and Vargas 2011, among others). Moreover, most of the vascular plant clades on the islands have a Mediterranean or North African origin (Francisco-Ortega *et al.* 1996; Carine *et al.* 2004; Marrero 2004; Kim *et al.* 2008). Although the colonization mechanisms and routes probably vary depending on the biological characteristics of each organism, the present and historical relative closeness of the Canary Islands to the potential source areas on the continent (e.g. Fuerteventura is currently ca. 116 km from Cape Juby-Tarfaya on the coast of Morocco, while 21,000 years BP they were separated by only ca. 65 km; Fig. 1) makes both recent and ancient long-distance dispersal (LDD) plausible, especially in plants with long-distance dispersal vectors. Even though the Canaries are oceanic (volcanic) islands, whose colonization is typically explained by long-distance dispersal events, the disjunct presence of Mediterranean elements in Morocco and the easternmost Canaries (i.e. Lanzarote and Fuerteventura, which are also the oldest extant islands) could be alternatively explained by other hypotheses considering the geographic closeness between the two areas (e.g. ‘stepping stones’ *sensu*Fernández-Palacios *et al.* 2011). Also, the currently separate islands of Lanzarote and Fuerteventura emerged initially as a single proto-island called Mahan, and the two islands were still joined as recently as the late Pleistocene (Fernández-Palacios *et al.* 2011).

The Alboran Sea in the westernmost Mediterranean to the east of the Strait of Gibraltar is a narrow basin (ca. 150 km wide and 350 km long at present) bordered on the north by the Baetic System (southern Spain). On the south it is bordered by the Rif (northern Morocco) mountain belts and by the South Balearic Basin to the east (Comas *et al.* 1992). In this area, the Quaternary climatic oscillations have partially moulded the genetic structure and spatial distribution of the biota and have led to speciation (Hewitt 1999). During the Last Glacial Maximum (LGM) the sea level was ~120–150 m lower than at present (Yokohama *et al*. 2000; Church *et al.* 2001; Clark and Mix 2002) and the Iberian and North African coast lines were closer. At that time, some of the submerged seamounts in the Alboran Sea could have emerged (Comas *et al.* 1992), thus facilitating the exchange of species between continents (Fig. 1). The genetic structure and diversity of several plant species has been heavily influenced by these dramatic geomorphological and environmental changes (e.g. Ortiz *et al.* 2007; Terrab *et al.* 2008a; Ortiz *et al.* 2009). However, currently only a few phylogeographic studies have focused specifically on herbs growing on both sides of the Alboran Sea (Silva *et al.* 2015).

Additionally, the Atlas Mountains may represent a formidable barrier for the migration of lowland xerophytic species, but its relative role in preventing such migrations has not yet been directly tested. This could be because North Africa is often under-represented in the surveys of Mediterranean taxa (Terrab *et al.* 2008a). An additional barrier for plant migration in Morocco is the Riffian Corridor, which today is occupied by the Loukos and Sebou river valleys that separate the Rif Mountains to the north from the Atlas ranges to the south. This corridor connected the pre-Mediterranean Sea with the Atlantic Ocean just before the Messinian Salinity Crisis (5.3 million years ago), and represented a strong barrier for the migration of plants both before and after the Messinian (Ortiz *et al.* 2009).

The focal species in this study is *Astragalus edulis*, an herbaceous annual Fabaceae (listed as Endangered in Spain) that lacks evident adaptations to LDD (Peñas 2004). It is restricted to grasslands on poor sandy soils that result from the erosion of volcanic or schistose rocks. This plant species grows in semi-arid ecosystems and currently occupies a highly disjunct distribution area (Peñas *et al.* 2016). It occurs in the semi-desert habitats of the south-eastern Iberian Peninsula, in the islands of Lanzarote and Fuerteventura, and in scattered locations in western North Africa (Morocco and Algeria), where it is distributed in three, disjunct population cores (Fig. 1). These population cores include one in north-eastern Morocco and north-western Algeria and two cores in south-western Morocco, the first one to the north of the High Atlas Mountains (steppes of El Haouz; Jahandiez and Maire 1931–34; Gómiz-García 2001) and the second one to the south of this mountain range (Sous plains and lowlands near the Anti-Atlas range; Gómiz-García 2001). The north-eastern and south-western population cores are roughly separated by the Rif and Middle Atlas Mountains.

Kay *et al.* (2006) have proposed that the genus *Astragalus* dates back 35 Ma, and M. F. Wojciechowski (pers. comm.) found that *A. edulis* diverged from its sister species *Astragalus boeticus* (Wojciechowski *et al.* 1999) significantly later, around 450–500 ka BP (based on ITS mutation rates). This suggests that the Messinian Salinity Crisis does not explain the present distribution of the study species. The strikingly disjunct distribution of *A. edulis* in the Iberian Peninsula, Morocco, and the Canary Islands therefore provides an ideal system to explore the postglacial evolutionary dynamics of a western Mediterranean endemic species present on both sides of the Alboran Sea and Atlas Mountains, which has also colonized the easternmost islands of the Canary archipelago.

This study seeks to reconstruct the phylogeographic patterns of intraspecific lineages within *A. edulis*, with the general aim of contributing to the understanding of the biogeographic history of the western Mediterranean–Macaronesian area. To do so, we will carry out the following: (i) address how the Mediterranean lineage *A. edulis* colonized the Canary Islands; (ii) infer the ancestral area of the species and explore possible colonization routes; and (iii) assess the role the Atlas Mountains have had as refuge areas for this species.

## Materials and Methods

### Amplified fragment length polymorphism data and analysis

An amplified fragment length polymorphism (AFLP) matrix corresponding to 360 individuals of *A. edulis* from Peñas *et al.* (2016) was used for this study.

The population genetic structure was examined using a Bayesian clustering method implemented in STRUCTURE v. 2.3.4 (Pritchard *et al.* 2000), following the approach described by Falush *et al.* (2007) for dominant markers. This method uses a Markov chain Monte Carlo simulation approach to group samples into an optimal number of *K* genetic clusters and does not assume the *a priori* assignment of individuals to populations or clusters. Analyses were based on an admixture ancestral model with correlated allele frequencies among populations (Falush *et al.* 2003). The proportion of membership of each individual and population to the *K* clusters was calculated performing 20 runs for each *K* value between 2 and 10 with a run length of the Markov chain Monte Carlo of 1 × 10^6^ iterations after a burn-in period of 1 × 10^6^ iterations. The optimal number of *K* clusters was estimated using the *ad hoc* parameter (Δ*K* statistic) of Evanno *et al.* (2005), as implemented in the online application of Structure Harvester software (v0.63; Earl and VonHoldt 2012).

### Plastid DNA sequencing and analysis

The plastid regions *trn*G*–trn*S, *trn*C*–rpo*B (Shaw *et al.* 2005) and *tab*F*–tab*C (Taberlet *et al.* 1991) were sequenced from 165 individuals from 17 species populations (Table 1). Haplotype variation was also explored using the information available for 61 individuals previously analysed by Peñas *et al.* (2016), using the same PCR conditions and primers for DNA amplification. PCR products were visualized on 1 % agarose gel and purified using the ExoSAP-IT PCR Clean-Up Kit (AFFIMETRIX, Santa Clara, CA, USA), following the manufacturer’s instructions. The cleaned amplicons were analysed using a 3730 DNA Genetic Analyser capillary sequencer (Applied Biosystems), and all sequences were deposited in GenBank. The total plastid DNA data set obtained from the 226 individuals was used (Table 1). Three samples of *A. boeticus* were used as the outgroup, based on the results of Wojciechowski *et al.* (1999).

**Table 1.** Locations, details and haplotypes for *Astragalus edulis.*

Population code	Locality	DIYABC metapopulations	Altitude	Longitude	Latitude	New individuals	Total cpDNA individuals	Haplotypes
AE1	Spain; Almería, Alcubillas	IP	735	−2,6025	37,0987	8	12	II and VII
AE2	Spain; Almería, Tabernas	IP	915	−2,4643	37,1306	7	13	I and VII
AE3	Spain; Almería, Gérgal	IP	720	−2,5254	37,1209	8	16	I and II
AE4	Spain; Almería, Gérgal, Arroyo Verdelecho	IP	648	−2,4704	37,1002	8	14	I, V and VII
AE5	Spain; Almería, Tabernas, Desierto de Tabernas	IP	621	−2,4863	37,0668	7	13	I, II and VII
AE6	Spain; Almería, Filabres, Rambla del Saltador	IP	541	−2,3610	37,1206	7	15	I
AE7	Morocco; La Oriental, between El-Aïoun and Tanarchefi	NM	919	−2,6016	34,4174	12	14	I
AE8	Morocco; Taza, Jebel Guilliz	NM	425	−3,3496	34,4669	12	14	I and II
AE9	Morocco; Marrakech, Chemaia, prox. Kettara	NA	480	−8,1875	31,8729	10	12	II and VII
AE10	Morocco; Marrakech, between Marrakech and Chichaoua	NA	380	−8,6185	31,5720	12	14	VIII
AE11	Morocco; Taroudant, between Tasgount and Ighil	SA	1437	−8,4832	30,1831	12	14	I, II, III and IV
AE12	Morocco; Taroudant, between Irherm and Tata	SA	1710	−8,4478	30,0467	13	15	I and II
AE13	Morocco; Taroudant, Tafraoute, Tizi- n-Tarakatine, prox. El Jebar	SA	1484	−8,8587	29,7376	12	14	II and VI
AE14	Morocco; Taroudant, between Tafraoute and Tleta-Tasrite	SA	1620	−8,9385	29,6354	3	6	II, VI and VII
AE15	Spain; Canary Islands; Lanzarote, Vega de Temuime	CI	159	−13,728	28,9337	14	16	IX
AE16	Spain; Canary Islands; Fuerteventura, Tiscamanita	CI	234	−14,033	28,3576	7	9	IX
AE17	Spain; Canary Islands; Fuerteventura, Barranco de Majada Blanca	CI	181	−13,986	28,2673	13	15	IX

The cpDNA sequences were assembled, edited and aligned using Geneious pro^™^ 5.4 (Drummond *et al.* 2012), and further adjustments and optimizations of the alignments were carried out manually. Since no incongruence among regions was found (branches with high support were compared among the regions), the sequences from the three regions were concatenated into a single matrix based on the assumption that the plastid forms a single linkage group. Gaps (insertions/deletions) longer than 1 bp (i.e. 10 and 3 pb in *trn*G*–trn*S) were coded as single-step mutations (one binary character added to represent the presence/absence of the gap). In addition, no inversion was found in the regions analysed. Mononucleotide repeats of different sizes were excluded given that they seem to be prone to homoplasy at large geographic scales (Ingvarsson *et al.* 2003).

An unrooted haplotype network was constructed to infer the genealogical relationships among haplotypes using the statistical parsimony algorithm (Templeton *et al.* 1992) as implemented in TCS 1.21 (Clement *et al.* 2000).

### Approximate Bayesian computation analyses with DIYABC

An approximate Bayesian computation (ABC) statistical approach was employed to analyse the plastid DNA using the software DIYABC v2.1 (Cornuet *et al.* 2014). The aim of this approach was to compare the different phylogeographic hypotheses that could be used to explain the present distribution of *A. edulis*. DIYABC allows the posterior probabilities of alternative scenarios to be tested by simulating a large number of data sets in each case. The logistic regression procedure (Fagundes *et al.* 2007) estimates the occurrence of each scenario among the simulated data sets that are closest to the observed data.

Based on the results from a previous study (Peñas *et al.* 2016), as well as the geographical distribution of the species, the five most likely metapopulations (Canary Islands, CI; Iberian Peninsula, IP; northern Morocco, NM; northern Atlas, NA; southern Atlas, SA; Table 1) were previously considered as a working basis for the DIYABC. A set of 34 plausible alternative scenarios was constructed in order to test all possible phylogeographical hypotheses with respect to the following items: (i) what is/are the ancestral metapopulation(s); (ii) what is the origin of the Canary Island populations; and (iii) to test for putative LGM refugial areas.

Prior distributions of the parameters were chosen as an initial approach with a large interval, due to the lack of ancestral information. Parameters were corrected after the first test (a list of all parameters and prior distributions used to model scenarios is summarized in Table 2). Population sizes were set equally in all cases except for founder events. Divergence times were unrestricted to allow the program to set the most likely value. The JC69 model of nucleotide evolution (Jukes and Cantor 1969) was chosen, and the uniform mutation rate was set to (10^–9^–10^–7^).

**Table 2.** DIYABC estimated parameters and codes.

Parameter	Parameter code	Prior distribution			Estimated parameters
		Type	Initial interval	Final interval	Mean
Population effective sizes of the IP group	NIp	Uniform	{10–100000}	{10–160000}	3,13E+04
Population effective sizes of the NM group	NNm	Uniform	{10–100000}	{10–40000}	2,46E+04
Population effective sizes of the NA group	NNa	Uniform	{10–100000}	{10–160000}	1,08E+05
Population effective sizes of the SA group	NSa	Uniform	{10–100000}	{10–120000}	8,73E+04
Population effective sizes of the CI group	NCi	Uniform	{10–100000}	{10–40000}	1,45E+04
Founder event for CI group	NCib	Uniform	{10–500}	{10–300}	6,79E+01
Time of founder event for CI group	t1	Uniform	{10–1000000}	{10–200000}	1,50E+05
Isolation time for NA	t2	Uniform	{10–1000000}	{10–30000}	2,46E+04
Divergence time among the Moroccan populations	t8	Uniform	{10–1000000}	{10–200000}	
Divergence time among the IP + NM + SA groups	t3	Uniform	{10–1000000}	{10–200000}	4,11E+03
Divergence time among the IP + NM + NA + SA groups	t4	Uniform	{10–1000000}	{10–200000}	
Divergence time between CI and NA	t6	Uniform	{10–1000000}	{10–200000}	
Divergence time between [CI + NA] and [IP + NM + SA] complex	t5	Uniform	{10–1000000}	{10–200000}	
Divergence time among all groups	t0	Uniform	{10–1000000}	{10–200000}	
Mean mutation rate	Mµ	Uniform	{10^−9^–10^−7^}	{10^−9^–10^−7^}	3,44E-09

One million data sets were simulated for each scenario (Cornuet *et al.* 2008, 2010). The best scenario was chosen by calculating the posterior probabilities of each one by performing a polychotomous weighted logistic regression on the 1 % of simulated data sets closest to the observed data set (Cornuet *et al.* 2008, 2010). Scenarios under 20% posterior probability (logistic regression procedure) were discarded. In the next step, the different probable scenarios were combined under each hypothesis, at which time 90 % of the scenarios were discarded and those receiving the greatest weights (five, plus null scenario) were selected. Subsequent distributions of parameters were evaluated under the best scenario using a local linear regression on the 1 % closest simulated data sets with a logit transformation (Table 2). Confidence in the choice of scenario was tested by evaluating Type I and Type II error rates (Cornuet *et al.* 2010). Similarity between real data and simulated data sets was assessed for the best scenario to test the model adequacy using the posterior distribution of the parameter values.

### Distribution modelling and LGM bathymetry

To model the current climatic suitability of *A. edulis* and project it into the LIG (130 ka BP) and LGM (21 ka BP), the Bioclim climatic layers available at www.worldclim.com were downloaded (Hijmans *et al.* 2005). All known localities of the species (Podlech 1988) were visited to confirm the presence of the plant and the plant was not found in Algeria. Correlation analysis among bioclimatic variables was performed. Afterwards, a hierarchical cluster analysis of these variables was carried out to identify groupings of correlated variables, and a threshold of 0.8 was set to avoid redundancy. One variable from each group was selected and the variance inflation factor (VIF) values (Marquardt 1970) were used to test multicollinearity through the ‘vif’ function of the ‘HH’ R package (Heiberger 2015). One variable was excluded from the ones with the highest VIF values, and this procedure was repeated until no variables remained with a VIF value greater than five. This information was combined with theoretical considerations to select the appropriate climatic variables for the modelling; three variables were finally selected. The climatic features that are suspected to have an influence on the ecology and range limits of *A. edulis* are temperature seasonality (bio4), precipitation of wettest quarter (bio16) and precipitation of driest quarter (bio17). All the climatic variables were rescaled to a grid cell resolution of 2.5 arc-minutes (the spatial resolution of the LGM data set) within the function ‘resample’ implemented in package ‘raster’ (Hijmans 2015). A non-metric multidimensional scaling was performed for visualizing the relative position of *A. edulis* populations within the ecological space and for checking for climatic differences between populations. This was achieved using the ‘metaMDS’ and ‘ordisurf’ functions of the R library ‘vegan’ (R Core Team 2012; Oksanen *et al.* 2013).

Systematic sampling was implemented to avoid sampling bias, as described in Fourcade *et al.* (2014). Afterwards, multiple scenarios were evaluated using the package ENMeval (Muscarella *et al.* 2014), which implements the maximum entropy algorithm (Phillips *et al.* 2006). These models were run with the L, LQ, H and LQH feature combinations used by Muscarella *et al.* (2014) and a regularization multiple from 0.5 to 4.0 by 0.5. The selected method was the leave-one-out strategy (jackknife) to compensate for the low number of presence records (Pearson *et al.* 2007). The area under the curve (AUC) and the Akaike information criterion (AIC) were used to evaluate the models; models with AUC above 0.75 are considered potentially useful, 0.80–0.90 good and 0.90–1.0 excellent (Elith 2002). The best model was selected using these criteria. The palaeodistributions (LGM and LIG) were generated by projecting the best model onto past scenarios using the package ‘raster’ (Hijmans 2015).

At the LGM, the Earth’s ocean levels were at their lowest point and extensive reaches of dry land were exposed along the continental coasts. Some analyses have substantially narrowed the uncertainties regarding total changes in ice sheets and sea level and their proxies, suggesting a net decrease in eustatic sea level at the LGM ranging from 120 to 135 m (Church *et al.* 2001; Clark and Mix 2002; Lambeck *et al.* 2014).

The present-day topographic and bathymetric data covering the area were extracted from the ETOPO1 to map in detail the past and current shorelines. This model was built from numerous global and regional data sets, and is available in ‘Bedrock’ (base of the ice sheets) versions (NOAA 2009).

## Results

### Population structure based on AFLP

**Figure 2.**
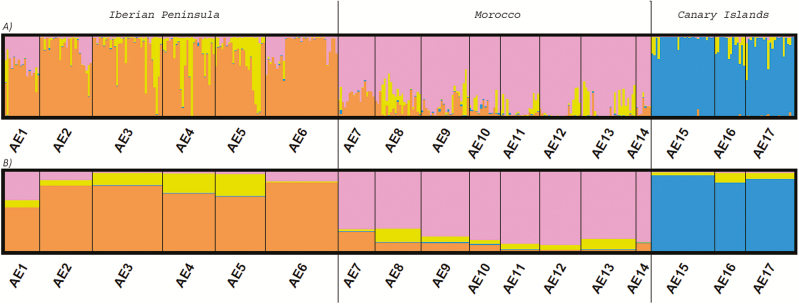
Results from the analysis of AFLP markers for *K* = 4. Histograms show the Bayesian clustering of individuals within populations, (A) admixture analysis, (B) population genetic structure.

Bayesian clustering conducted using STRUCTURE resulted in a best partition of four clusters with a maximum modal value of Δ*K* = 249.02 **[see****Supporting Information—Fig. S1****]**. Placement of the individuals within the different clusters is shown in Fig. 2. Individuals within Cluster A (orange) were found to be prevalent in the large metapopulation from south-eastern Spain and present in some of the Moroccan populations; individuals within Cluster B (pink) were dominant in all the Moroccan populations and displayed a significant presence in some populations from the Iberian Peninsula (i.e. AE1, AE2 and AE6); individuals within Cluster C (blue) were dominant in the Canary Islands and residual in the other groups; and individuals within Cluster D (yellow) were present (although never dominant) in almost all species populations.

### Chloroplast variation and geographical distribution of haplotypes

The length of the three cpDNA regions in the recently collected 165 individuals, plus the 61 taken from Peñas *et al.* (2016), ranged from 630 and 772 bp and resulted in a final alignment of 2092 bp. In the *trn*G–*trn*S region, three polymorphisms (two indels/one substitution) were detected across the whole data set, while four substitutions and one substitution for *trn*C*–rpo*B and *tab*C*–tab*F were found, respectively.

All mutations together defined a total of nine haplotypes (Table 1). TCS inferred a 95 % parsimony network with a maximum limit of five steps (Fig. 1). Intrapopulational haplotype variation was detected in 11 sampling sites (AE1, AE2, AE3, AE4, AE5, AE8, AE9, AE11, AE12, AE13 and AE14; Table 1; Fig. 1). The most frequent haplotype (I) was found in five sampling sites from the Iberian metapopulation, in the north-eastern Moroccan populations and in AE11 and AE12 from south-western Morocco. The second most frequent haplotype (II) was represented in five populations from south-western Morocco, in one from north-eastern Morocco and in three sampling sites from the Iberian Peninsula. The large Iberian metapopulation contained one endemic haplotype (V) and the south-western Moroccan populations contained four endemic haplotypes (III, IV, VI and VIII). A single endemic haplotype (IX) was found in Fuerteventura and Lanzarote.

### Modelling of plausible demographic scenarios and estimated times of divergence

**Figure 3.**
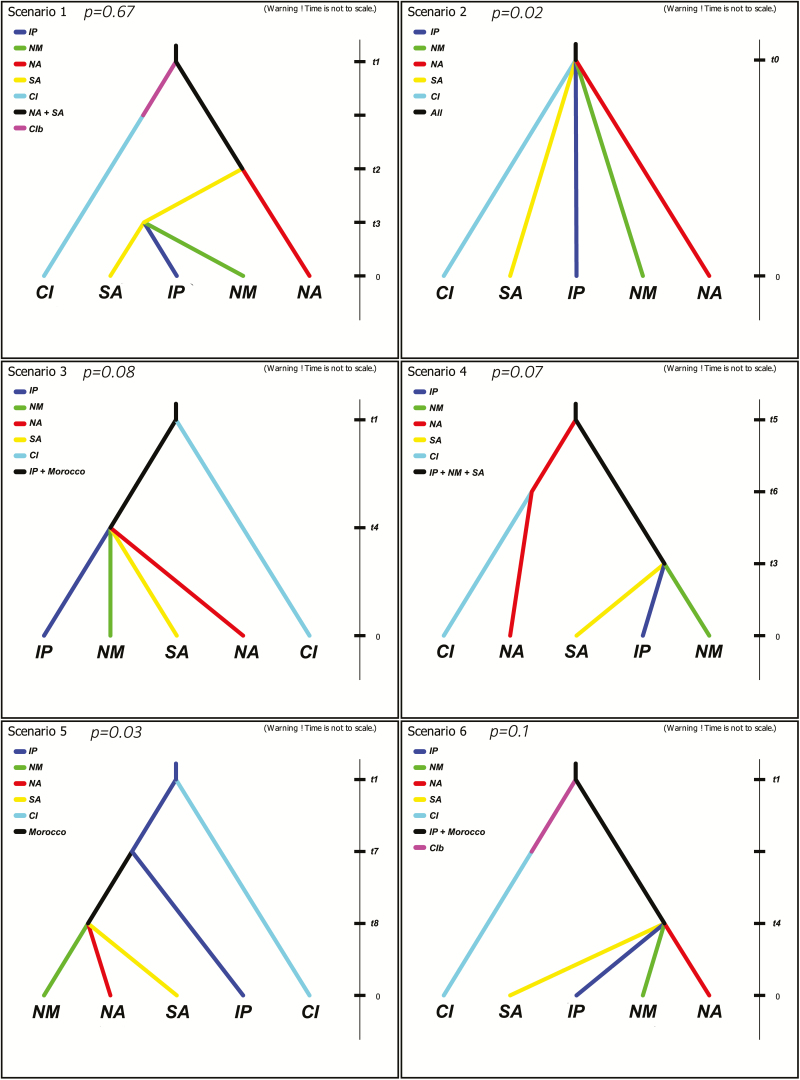
Approximate Bayesian computation analysis of *Astragalus edulis*. Most likely DIYABC scenarios (posterior probability is shown); Time is not to scale; Areas (southern Atlas, SA; northern Atlas, NA; northern Morocco, NM; Iberian Peninsula, IP; Canary Islands, CI; Canary Islands founder event, CIb).

Here, only the six most plausible scenarios are shown (Fig. 3). The scenario with the highest posterior probability was Scenario 1 (*P* = 0.6799 [0.6703–0.6849]) followed by Scenario 6 (*P* = 0.1074 [0.1014–0.1134]), Scenario 3 (*P* = 0.0878 [0.0826–0.0929]), Scenario 4 (*P* = 0.0733 [0.0680–0.0786]), Scenario 5 (*P* = 0.0303 [0.0282–0.0325]) and Scenario 2 (*P* = 0.0214 [0.0194–0.0234]). The best scenario consisted of an early founder event from Morocco mainland to the Canary Islands, which occurred ca. 150000 (127000–173000) generations ago, before the end of the Riss glaciation and when Lanzarote and Fuerteventura were still joined together (Fernández-Palacios *et al.* 2011). This lead to the establishment of an initial population followed by an expansion and colonization of the area, with increasing population sizes (Table 2). According to this scenario, the next evolutionary event would have been the isolation of the NA metapopulation (ca. 24600 generations ago), while the groups of populations from IP and NM would have diverged from those in SA ca. 2400 generations ago. These data support SA + NA as the original ancestral area.

The Type II error rate, which is the probability that data sets simulated under other scenarios were assigned to the best scenario, was 20 %. The Type I error rate, the probability that data sets simulated under the best scenario were assigned to other scenarios, was 44 %, which may be due to high similarities among scenarios. The similarity between real data and simulated data sets for the best scenario was calculated **[see****Supporting Information—Fig. S2****]**, and it was found that from a total of 13 summary statistics only one case of statistics diverged from the simulated ones (*P*-value < 0.05).

### Distribution modelling

**Figure 4.**
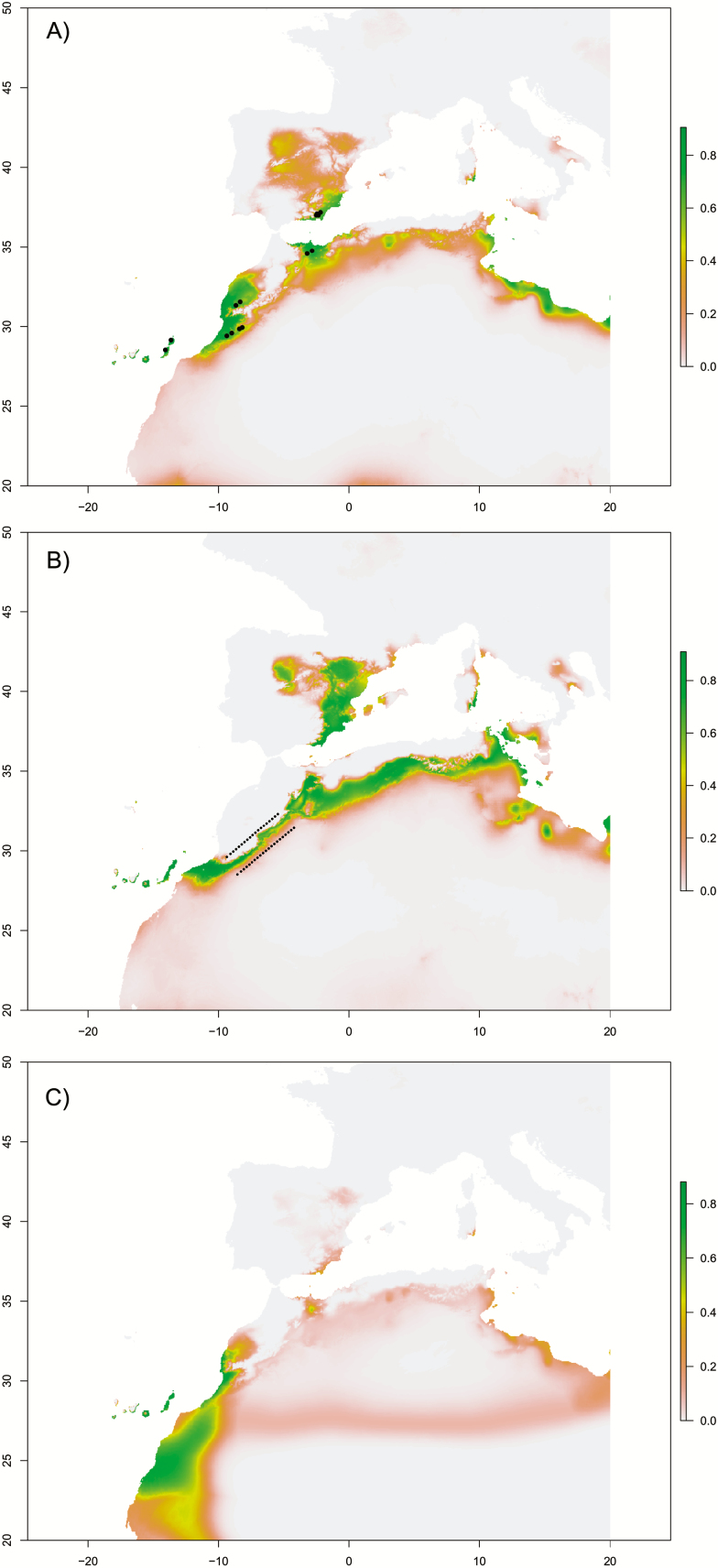
Distribution models; habitat suitability is represented by green-yellow to red (red-yellow = medium, green = high). (A) Present; (B) LGM; (C) LIG. Dotted line represents the south to north colonization route along Morocco. Black dots represent current localities of the species.

The model corresponding to the potential present distribution of the species (Fig. 4A) showed high predictive accuracy (AUC = 0.98). The currently known distribution of the species mostly coincided with that predicted by the model (Fig. 4A). From the three bioclimatic variables used in the analyses, bio16 showed the highest explanative power (relative variable contribution 67 %). The past suitable areas for the species in the LGM and LIG are shown in Fig. 4B and C, respectively. They included a continuous corridor (Fig. 4B) that extended along the south of the Atlas Mountains to the north-eastern part of Morocco (with high suitability values in the area of the Moulouya river valley) during the LGM. During this period, an area of high suitability was also found to the east of the Iberian Peninsula (south of the Pyrenees). The model projected to the LIG period showed only two suitable areas for the species: a mainland area along the Atlantic coast at the westernmost edge of the Atlas Mountains, corresponding to the westernmost extremes of population groups SA and NA; and the eastern Canary Islands. Lastly, the extent of the potential area suitable for the species *A. edulis* appears to have been at its largest during the LGM.

The estimations of emerged land area at LGM, with respect to the present-day, are the result of raising the values of the digital elevation model 120 m (Fig. 1).

## Discussion

### Ancient colonization of the Canary Islands by *Astragalus edulis*

It is generally accepted that all lineages on oceanic islands originated from mainland lineages through long-distance dispersal events (MacArthur and Wilson 1967; Ganders and Nagata 1984; Baldwin and Robichaux 1995; Poulakakis *et al.* 2012). The establishment of *A. edulis* in the Canaries could be a result of either a recent or ancient long-distance dispersal event or a combination of the two, as a consequence of multiple dispersal events that occurred at different times.

As a general pattern, due to founder events and restricted gene flow, lower levels of genetic variation are expected to be found on islands than in their mainland counterparts (Frankham 1997; Baldwin *et al*. 2008), although at least one exception is known (Fernández-Mazuecos and Vargas 2011). Additionally, in long-term isolated populations the rarity value (frequency down-weighted marker value, DW) is expected to be high, because rare markers should accumulate due to mutations. Newly established populations, on the other hand, are expected to exhibit low rarity values, and thus help in distinguishing old vicariance from recent dispersal (Schönswetter and Tribsch 2005). Putative refuge areas are typically characterized by high genetic distinctiveness (DW), as well as by high genetic diversity, while long-distance dispersal events can be recognized by comparatively low values of rarity and genetic diversity. The AFLP data suggest that limited gene flow exists among the populations from the Canary Island and the Moroccan or Iberian populations (Fig. 2). This is corroborated by the neighbor joining (NJ) and principal coordinate analysis (PCoA) analyses performed, based on AFLP data (Peñas *et al.* 2016), and suggests long-term isolation of the Canarian populations. Additionally, the genetic diversity and rarity values found do not support recent long-distance dispersal events from Morocco (mean Nei’s diversity index 0.1013 in the Canaries vs. 0.1331 in the remaining distribution area of the species; mean DW 4.199 in the Canaries vs. 2.888 in the remaining distribution area of the species). These data would support long-term *in situ* survival of *A. edulis* in the Canaries or simple ancient LDD followed by isolation, which is consistent with the haplotypic diversity pattern (Fig. 1). The hypothesis of multiple LDD events or recurrent contact between the Moroccan and Canarian populations was not supported by our data, since high levels of gene flow between Morocco and the Canary Islands were not found. These results are further supported by the almost negligible admixture degree detected between the populations of the Canary Islands and any of those from the mainland areas (Fig. 2A).

Regarding plastid DNA, the well-supported close relationship between haplotypes VIII (exclusive to population AE10) and IX (endemic to the Canary Islands; Fig. 1) indicates connections between Morocco and the Canary Islands. Western Morocco, particularly to the north of the High Atlas range, appears to be the primary source area for the initial colonization of the islands. Moreover, the best-supported phylogeographic scenario, as detected by the DIYABC, involves a single ancient LDD founder event (150 ka BP) from the Atlas (NA + SA) area to Mahan, followed by colonization of the area. The age of the inferred LDD event is in concordance with the diversity and DW values obtained. Although *A. edulis* lacks evident adaptations to long-distance dispersal, the Moroccan coast and the eastern Canary Islands are relatively close. Also, the falling sea level during the Riss glaciation would have promoted the emergence of previously submerged seamounts that could have acted as stepping stones to facilitate floristic interchanges between these regions (Fernández-Palacios *et al.* 2011). The AFLP data indicate that Fuerteventura was probably colonized first, given the high levels of diversity and rarity (Peñas *et al.* 2016). This would be consistent with the present and historical (particularly during the glacial maxima) proximity between populations AE16 and AE17 and Cape Juby-Tarfaya in Morocco.

The phylogeographic relationships of *A. edulis* indicate that the inter-island colonization between similar ecological zones found for other plant species (e.g. Francisco-Ortega *et al.* 1996; Fernández-Mazuecos and Vargas 2011) is not, in this case, the mechanism for establishing populations on different islands. Postglacial colonization between Fuerteventura and Lanzarote is not supported by our results, since the populations collected on the two islands share the same Canarian endemic haplotype. Additionally, the overall genetic composition, as revealed by AFLP data, is highly homogeneous, which is congruent with the fact that the currently separate islands emerged as a single proto-island (Mahan) and remained joined together as recently as the late Pleistocene (Fernández-Palacios *et al.* 2011).

### South-western Morocco as ancestral area for *Astragalus edulis* and subsequent migration to the north-east

Palaeodistribution models (Fig. 4C) showed the existence of an area, located to the north and south of the westernmost edge of the High Atlas mountain range, which was highly suitable for the species during the LIG. The coalescent-based ABC method, as implemented by the DIYABC software, also identified this area (metapopulations northern Atlas and southern Atlas) as ancestral for the species. A similar ancestral area has been found for other annual herbs (e.g. *Hypochaeris arachnoidea*, Ortiz *et al.* 2009). This is also consistent with the haplotype network, which shows haplotype II in a central, probably ancestral, position.

DIYABC also identified an isolation of metapopulation northern Atlas around 24600 generations ago (probably near the LGM) and a subsequent colonization to the north-east from southern Atlas to northern Morocco and Iberian Peninsula. Accordingly, the palaeogeographic models show a corridor in terms of suitable habitat for the species during the LGM along the southern slopes of the Anti-Atlas, High Atlas, and Tell Atlas (Hamada desert habitat) connecting to the north with the Moulouya river valley (Fig. 4B).

The Bayesian modelling of demographic scenarios supports the contention that the divergence between northern Morocco and Iberian Peninsula took place very recently (2400 generations ago). Although this is not supported by the AFLP data, as populations on both sides of the Alboran Sea form distinctive AFLP clusters in the NJ and PCoA analyses (Peñas *et al.* 2016), some degree of admixture has been identified by the STRUCTURE analysis (Fig. 2). Additionally, the central haplotypes I and II, plus haplotype VIII are shared among many populations from the Iberian Peninsula and Morocco and only haplotype V is endemic to the Iberian Peninsula. Notably, both the present and the palaeodistribution models consistently suggest that the area of the Strait of Gibraltar, which was involved in the exchange of species between North Africa and the Iberian Peninsula (e.g. Rodríguez-Sánchez *et al.* 2008; Lavergne *et al.* 2013), presents no appropriate habitat for *A. edulis*. However, both sides of the Alboran Sea have historically presented conditions that are suitable for the species (Silva *et al.* 2015).

It also bears noting that many plants, such as *Caralluma munbyana* (Asclepiadaceae), *Launaea arborescens* (Asteraceae), *Logfia clementei* (Asteraceae), *Lycium intricatum* (Solanaceae), *Maytenus senegalensis* (Celastraceae), *Notoceras bicorne* (Brassicaceae), could have also followed this colonization route from south-east Morocco to the Iberian Peninsula through the area of the Alboran Sea.

### The role of the High Atlas mountain range in shaping the genetic diversity of *Astragalus edulis*

Few studies have focused on the role of the High Atlas as a barrier to gene flow for annual and perennial herbs (e.g. Ortiz *et al.* 2009). Moreover, to date, very little is known about the Quaternary range dynamics of plant species in the area and precise locations of refugia frequently remain unknown (Terral *et al.* 2004; Rubio *et al.* 2006). Regarding annual herbs, the existence of refuge areas at low altitudes around the Atlas Mountains has been proposed for plants such as *H. arachnoidea* (Ortiz *et al.* 2009), *Hypochaeris angustifolia* (Terrab *et al.* 2009) and *Arabidopsis thaliana* (Brennan *et al.* 2014).

In the case of *A. edulis*, the AFLP data analysed (Fig. 2) showed no evidence of the High Atlas Mountains acting as a barrier to gene flow, but these results may underestimate the importance of this mountain range. The maintenance of endemic haplotypes (haplotype VIII to the north of the High Atlas and haplotypes III, IV and VI, to the south of this mountain range) suggests long-term isolation of populations at low altitudes. This idea is also supported by the early isolation of the northern Atlas metapopulation group as detected by the DIYABC analysis. Thus, our data appear to confirm the presence of low altitude refuge areas for annual species at favourable locations around the area of the High Atlas and Anti-Atlas mountain ranges. These locations could represent additional ‘phylogeographical hotspots’ (Médail and Diadema 2009), which are ‘significant reservoirs of unique genetic diversity favourable to the evolutionary processes of Mediterranean plant species’.

## Conclusions

Our results suggest that the populations of *A. edulis* on the Canary Islands are the consequence of an ancient LDD event, probably from the western Moroccan populations during the Riss glacial stage. Moreover, our results indicate that the original area for the species is located in the western part of the High Atlas Mountains. A colonization route is proposed that connects the southern Atlas region with the region that is currently occupied by the northern Moroccan populations of *A. edulis*, which finally reaches the Iberian Peninsula. This route may have also been followed by other plant species, some of which are also endangered and with fragmented distributions.

## Sources of Funding

This work has been financed by the Spanish Ministerio de Ciencia e Innovación through the projects CGL2012-32574 and REN2003-09427, as well as by the Andalusian Consejería de Innovación, Ciencia y Tecnología through the project RNM1067. The funders had no role in study design, data collection and analysis, decision to publish or preparation of the manuscript.

## Conflict of Interest

None declared.

## Contributions by the Authors

J.B.-P. performed the experiments, analysed the data, contributed reagents/materials/analysis tools, wrote the paper, prepared figures and/or tables, reviewed drafts of the paper. J.P.d.G. conceived and designed the experiments, contributed reagents/materials/analysis tools, wrote the paper, reviewed drafts of the paper. N.L.-G. analysed the data, prepared figures, reviewed drafts of the paper. S.M. contributed reagents/materials/analysis tools, reviewed drafts of the paper. M.M.M.-O. conceived and designed the experiments, analysed the data, contributed reagents/materials/analysis tools, wrote the paper, reviewed drafts of the paper.

## Supplementary Material

Supplementary_Material

## Supporting Information

The following additional information is available in the online version of this article—

**Figure S1.** Graph of delta *K* values to determine the ideal number of groups.

**Figure S2.** Principal coordinates analysis showing the accuracy of the best scenario, as determined by DIYABC.
