# Supplementary material for: Phylogeography of an endangered disjunct herb: long-distance dispersal, refugia and colonization routes
Source: AoB Plants. 2018 Aug 21;10(5):ply047. doi: 10.1093/aobpla/ply047 (PMC6150253; doi:10.1093/aobpla/ply047)
Supplement: Supplementary_Material [file ply047_suppl_supplementary_material.pdf]

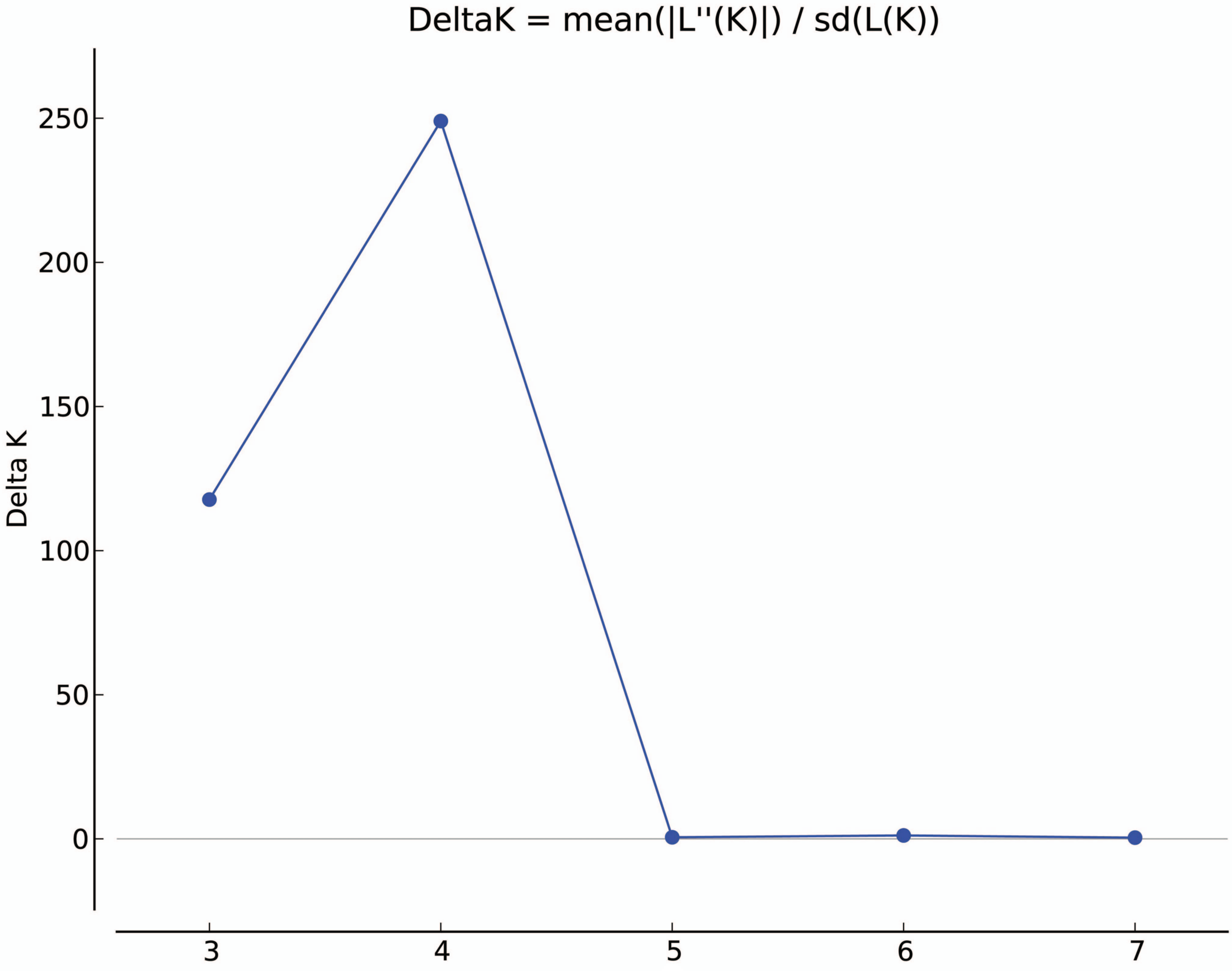

Figure S1: Graph of delta K values to determine the ideal number of groups.

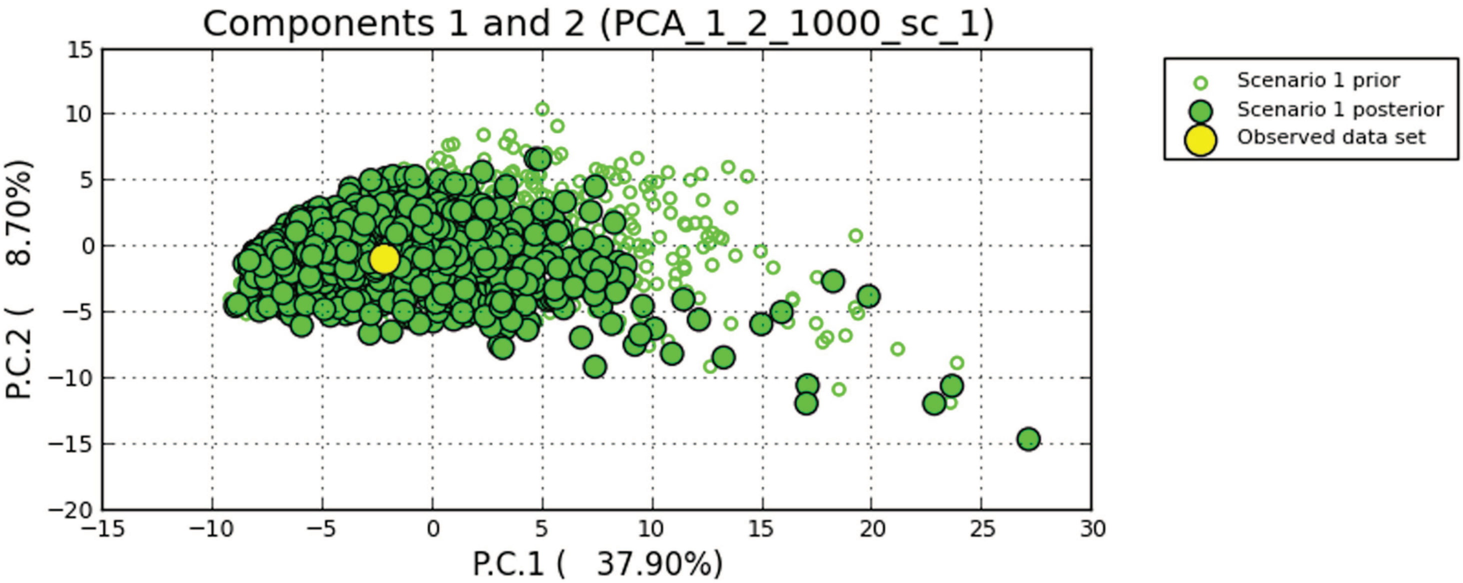

Figure S2: Principal Coordinates Analysis showing the accuracy of the best scenario, as determined by DIYABC.
